# Maffucci’s syndrome in association with giant tubular adenoma of the breast: Case report and literature review

**DOI:** 10.1016/j.ijscr.2019.09.012

**Published:** 2019-09-23

**Authors:** Dennis Mazingi, Chenesa Mbanje, George Jakanani, Godfrey I. Muguti, Valentine Mandizvidza, Shingi Bopoto

**Affiliations:** a College of Health Sciences, University of Zimbabwe, Parirenyatwa Hospital, Mazowe Street, P.O. Box A168, Avondale, Harare, Zimbabwe; b Diagnostic Imaging Centre, 17 Lanark Road Belgravia, Harare, Zimbabwe

**Keywords:** Case report, Tubular adenoma, Maffucci syndrome, Breast, Enchondromatosis

## Abstract

**Introduction:**

Maffucci’s syndrome is a rare congenital, non-hereditary mesodermal dysplasia characterised by multiple enchondromas and vascular lesions. To our knowledge this is the first report of tubular adenoma of the breast in association with Maffucci’s syndrome.

**Presentation of case:**

We report a 31-year-old female who presented with a large, ulcerated slow-growing painless breast mass. She had also sustained two pathological fractures during childhood as well as progressive deformity and limb shortening on the right side of the body. Skeletal survey revealed enchondromas exclusively on the right side in association with phleboliths. Excision of the breast mass was performed. Histological examination of the breast lesion revealed a diagnosis of tubular adenoma, core biopsy of the bony lesions confirmed enchondromas and a cutaneous haemangioma was excised from the abdominal wall.

**Discussion:**

Tubular adenoma of the breast is a rare benign tumour. When large they may cause ulceration leading to diagnostic and management dilemmas. Patients with Maffucci’s Syndrome require lifelong surveillance for the development of malignancy.

**Conclusion:**

Tubular adenomas of the breast carry no risk of malignancy. However, malignancy remains high on the differential list when the skin is ulcerated. Ulceration can also be caused by pressure necrosis in benign tumours. This diagnostic dilemma can lead to unnecessarily radical surgery.

## Highlights

• Maffucci’s syndrome is a rare congenital musculoskeletal disorder.
• Life-long surveillance is mandatory for early detection of malignancies.
• Tubular adenoma of the breast is benign with an almost non-existent risk of malignant transformation.
• Benign breast tumours may cause ulceration of the skin leading to diagnostic confusion with malignancy.
• Giant tubular adenomas of the breast may be treated with lumpectomy even when the ratio of abnormal breast tissue to normal breast tissue is very high.

## 1 Introduction

This case has been reported in accordance with the surgical case report guidelines (SCARE) criteria [1]. Maffucci’s syndrome is a congenital, non-hereditary mesodermal dysplasia characterized by multiple enchondromas and vascular lesions [2]. Affected patients suffer from physical deformity resulting in aesthetic and functional sequelae and as well as pathological fractures. They also have a significant lifetime risk for the development of various skeletal and non-skeletal malignancies [3]. Tubular adenoma of the breast is a rare benign breast neoplasm that has an almost non-existent risk of harbouring malignancy [4].

We report a case of giant tubular adenoma of the breast and Maffucci’s syndrome in a 31-year-old female patient whom we managed at our teaching hospital. The case presented below is unique because to our knowledge this is the first report of giant tubular adenoma of the breast in a patient with Maffucci syndrome. We emphasise the need for physicians to recognise this rare clinical entity because of the need for lifelong surveillance. We also show that while a high index of suspicion for breast malignancy should be maintained when there is ulceration and lymphadenopathy, in rare cases there are plausible alternative explanations. Our experience in this case shows that surgeons should attempt to conserve the breast even when very large tumours replace most of the normal breast tissue.

## 2 Case report

A 31-year-old female patient was referred to our general surgical unit by a general practitioner with a large, painless right-sided breast mass which had been growing slowly for the last 10 years. She presented with an ulcer on the same breast that began as a hot porridge scald burn 15 months before. The ulcer had since increased in size and become fungating. During her childhood, she had been of the same stature as her peers. However, her growth had started to lag around the age of 5 years. Around the same age, she had noticed small nodular growths which had begun to appear on the fingers of her right hand as well as angular deformity of the right upper limb and right lower limb. A few months later, she sustained a fracture of her right tibia after a trivial fall as well as another pathological fracture a year later at the shoulder region. Over the rest of her childhood and adolescence, her limb deformity worsened. She was otherwise in good general health. She was right-handed however, the hand deformity did not prevent her from using that hand for daily tasks or writing. She was also able to walk, albeit with a limp and she reported a minimal effect on her work as a subsistence farmer. She had no history of oral contraceptive use and had experienced menarche at the age of 14 years. There was also no family history of breast neoplasms. None of her 5 siblings nor her parents or grandparents were affected by a similar condition of the limbs. Apart from this, her drug, family and psychosocial histories were non-contributary.

**Fig. 1:**
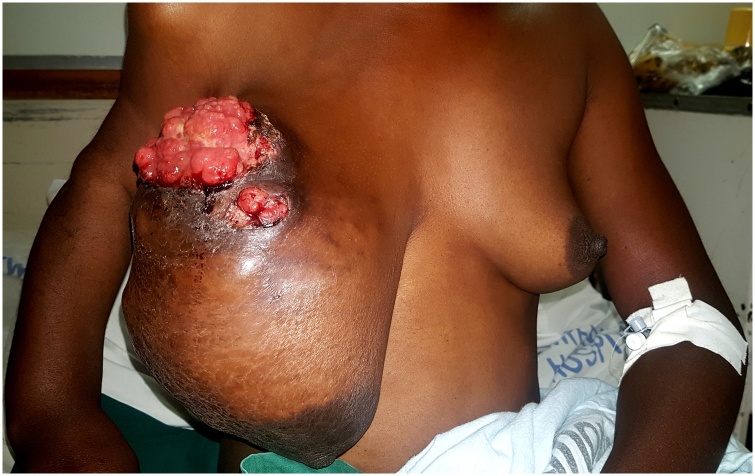
Preoperative appearance of breast mass. Image of the breast lesion illustrating the superolateral fungating breast ulcer on a large, irregularly shaped, nodular breast mass.

**Fig. 2:**
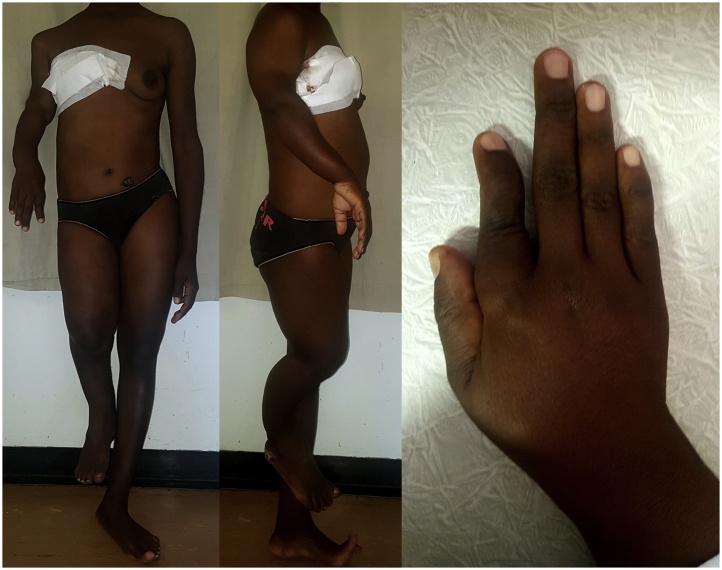
Examination findings. 2A: an anterior image of the patient illustrating the limb deformity confined to the right side of the body with limb shortening and irregular angular deformity. 2B: a lateral image of the patient illustrating limb abnormalities. 2C: Image of the patient’s right hand illustrating nodular prominences of the bones of the hand and fingers as well as finger shortening and irregular angular deformity.

Examination revealed a well-circumscribed, irregularly shaped, firm, mobile breast mass, approximately 15 cm in its transverse diameter and 20 cm in its superior/inferior diameter replacing >90% of the normal breast tissue. There was associated solitary, mobile ipsilateral lymphadenopathy. There was a superolateral fungating ulcer which was friable and surrounded by inflammatory skin changes and downward nipple displacement (Fig. 1). She was of short stature (149.5 cm), with unilateral distribution of bony abnormalities on the right side. She had irregular angular deformity and limb shortening (Fig. 2A and B) of the upper and lower limb, causing a limp favouring the right side as well as shortening and deformity of the thumb and index finger (Fig. 2C) with associated nodularity. She had no facial dysmorphic changes. She also had a pedunculated, soft hyperpigmented mass on the anterolateral abdominal wall, which was 2 × 2 cm in size (Fig. 2A).

A core needle breast biopsy revealed findings consistent with a tubular adenoma. Because of the breast ulceration and solitary ipsilateral lymph-nodes considered to be suspicious for malignancy, two repeat core needle biopsies were performed and examined by independent pathologists which both confirmed the initial result. The patient was prepared for theatre. Preoperative assessment included a skeletal survey.

**Fig. 3:**
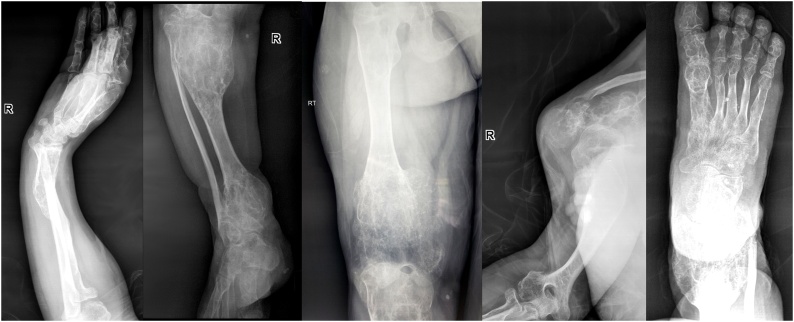
**A, B, C, D, E**. X-ray images of the patient’s bony abnormalities. 3A: Radiographs of the right hand illustrating multiple enchondromas in the phalanges and metacarpals. 3B: Radiographs of the right leg, 3C: right hip, thigh and knee, 3D: right shoulder and arm, 3E: right foot showing multiple enchondromas of the long bones confined to the right side of the body. A calcified phlebolith is visible in the periarticular soft tissues of the right knee joint.

Radiographs of the right hand revealed multiple enchondromas in the phalanges and metacarpals (Fig. 3A), as well as in the long bones confined to the right side, (Fig. 3B–E). Typical “ring and arc” chondroid type calcifications were associated with these lesions. Multiple well defined calcified phleboliths were identified within the soft tissues, and these were indicative of associated haemangiomata. The radiographic abnormalities were deemed classical for Maffucci’s syndrome

A simple mastectomy was performed for removal of the mass because almost the entirety of the breast tissue was replaced by the tumour. Two enlarged lymph nodes were noted and also removed. The procedure was performed by a junior resident with 4 years of specialised training under the supervision of a qualified, experienced surgeon. Her post-operative course was uneventful, she did not experience any complications of surgery. The mastectomy specimen was submitted for pathological examination. Her pedunculated abdominal skin lesion was also removed and sent for pathological examination as well as core biopsies of the cystic bony masses noted on x-ray.

**Fig. 4:**
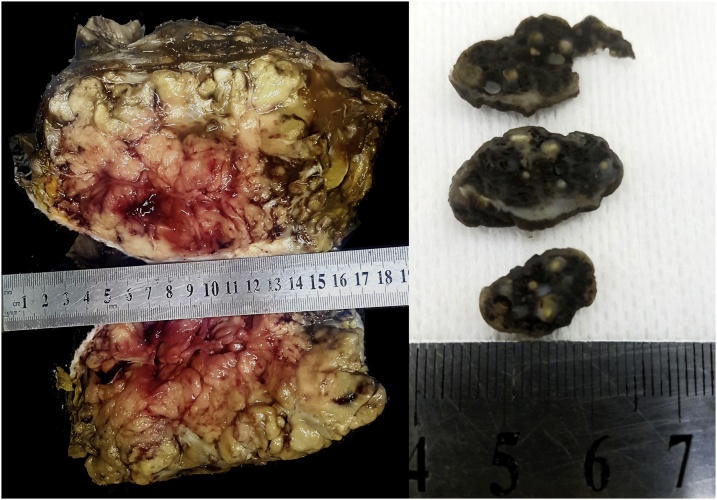
**A, B.** Images of gross pathological specimens. 4A: gross specimen of the excised breast showing the dimensions of the tubular adenoma and the well circumscribed mass with a capsule. 4B: images of the gross pathological specimen of the pedunculated skin lesion illustrating phleboliths shown as rounded concretions within the dark soft tissue containing altered blood.

**Fig. 5:**
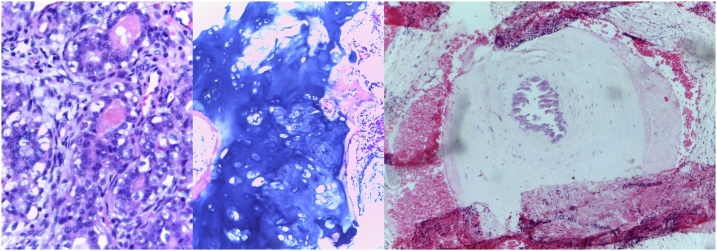
**A, B, C.** Images of the histopathological features of the lesions which were a component of the patient’s condition. 5A: Illustrates the tubular adenoma at x40 magnification showing lobules of bland tubular glands lined by 2 layers of cells with a surrounding capsule. 5B: shows histopathological features of the enchondromas from a core biopsy at the distal tibia at x10 magnification. 5C: shows histopathological features of the cavernous haemangioma from the skin lesion with a phlebolith in the capillary lumen at ×10 magnification.

Pathological examination of the mastectomy sample revealed a large, well-circumscribed breast mass with a well-defined capsule (Fig. 4A). The tumour was 18 cm × 15 cm × 10 cm with minimal, compressed normal breast tissue adjacent to it. The tumour was composed of lobules of bland tubular glands lined by two layers of cells with a surrounding capsule. The trabeculae were dense and fibrous with focal calcifications (Fig. 5A). The overlying skin was ulcerated with an associated dense mixed inflammatory infiltrate and focally acantholytic epidermis. A diligent search was made throughout the tumour for malignancy but none was found. Of the lymph nodes sampled, none showed any tumour, and all showed follicular lymphoid hyperplasia. Pathological examination of the skin lesion (Fig. 4B) noted a dermal lesion composed of cystically dilated blood vessels with marked congestion, thrombosis and focal calcification in keeping with a cavernous haemangioma. Core bone biopsy of the bony lesions noted lobules of benign cartilage encased by spongy bone marrow consistent with enchondroma with no evidence of malignancy seen (Fig. 5B).

Given the above pathological features, a final diagnosis of Maffucci’s syndrome with giant tubular adenoma was made. The patient did not wish to have surgery of her bony deformities for cosmetic or functional reasons. Her primary concern had been the large breast mass which had attracted stigma from members of her community and we achieved a high degree of patient satisfaction. On 1-year follow-up, the patient is free of malignant disease and had experienced no postoperative complications. A plan was made for surveillance every year with clinical examination for early detection of occult malignant tumours. A timeline of events is provided in Table 1.

**Table 1:** Timeline of events in patient’s history.

Age of patient	Event
5 years	Bony deformity and growth slow-down becomes apparent
5 years, 8 months	Patient experiences a pathological fracture (right tibia)
6 years, 9 months	Patient experiences another pathological fracture (right shoulder area)
21 years	Noticed a slow growing right-sided breast mass
29 years	Sustained a porridge burn on the right breast
31 years	Presented to our unit
	Postoperative follow-up (1 week later)
32 years	1 year follow-up
33 + years	Continued follow-up

## 3 Discussion

Maffucci’s syndrome was first described in 1881 by the Italian pathologist Angelo Maria Maffucci [5]. It is a rare condition with approximately 200 cases having been described as of 2016 [3]. It is characterised by multiple enchondromas (benign cartilaginous tumours) and vascular lesions. The enchondroma lesions are typically found at the metaphysis of the long bones, commonly in the hand, foot, femur, tibia, and fibula but it may also affect flat bones such as the pelvis [6]. They are unilaterally distributed in 50% of patients [7]. It is a non-hereditary condition with no sex or race predilection. The syndrome becomes clinically overt from birth up to the first year of life in 25% of cases. By the age of six years, 45% of cases have manifested and by puberty, this number has increased to 78% [8].

The feature which distinguishes Maffucci syndrome from the closely related Ollier’s disease is the presence of vascular lesions, particularly cavernous haemangiomas. Haemangiomas may appear as subcutaneous nodules or as visceral vascular lesions. They may also appear as phleboliths on radiographs in close association with the enchondromas representing soft tissue calcifications in the haemangiomas. These are apparent in our case. Our patient also had a cutaneous haemangioma. Multiple neoplasms have been reported in patients with Maffucci syndrome including breast carcinoma and fibroadenoma [8], however, to our knowledge tubular adenoma of the breast has not previously been described with this entity.

Management of enchondromas includes treatment for pathological fractures, surgical correction of disfiguring deformity and excision for symptomatic lesions or malignant transformation. Regular surveillance is important to detect the development of skeletal malignancy as a result of malignant degeneration of the enchondromas as well as the multiple other non-skeletal neoplasms that are almost inevitable in these patients [9].

Tubular adenomas are rare benign neoplasms of the breast that account for 0.13–1.7% of all benign breast lesions [10]. Adenomas of the breast comprise a spectrum of disease depending on the preponderance of glandular tissue. Tubular adenomas are thought to be a variant of pericanalicular fibroadenoma with an exceptionally prominent or florid adenosis-like epithelial proliferation [12]. The characterisation of this entity has been fraught with difficulty and some controversy stemming from its similarities to other related pathologies and lack of consensus regarding naming. Hertel, in 1976 attempted to classify these related breast lesions as follows [13] based on morphological criteria (Table 2).

**Table 2:** Hertel’s classification of breast adenomas [13].

I.	True adenomas
	a. Tubular adenoma
	b. Combined tubular and fibroadenoma
	c. Lactating adenoma
	d. Sweat-gland tumours; eccrine acrospiroma; eccrine spiradenoma
II.	Nipple adenoma
III.	Fibroadenoma

While the morphological features of these tumours are well characterised, their genetics are only recently coming to light. In a genetic study of various breast lesions, mutated tubular adenomas showed primarily mutations in MET and FGFR3 [11], whereas enchondromas in Maffucci’s syndrome have been known to harbour isocitrate dehydrogenase (IDH1 and 2) mutations [8]. The simultaneous occurrence of these conditions does not infer association particularly when a plausible genetic link has yet to be discovered. Future genome-wide association studies may one day help elucidate if any aetiological link exists between Maffucci’s syndrome and its many associated neoplasms.

Tubular adenomas of the breast are considered benign tumours with a virtually non-existent risk of malignant transformation [12]. They usually range in size from 1 cm to 7.5 cm [14]. To our knowledge, this is the largest tubular adenoma yet described in the literature at 18 cm in its largest diameter. The largest tumour reported previously in the literature was a 15 cm tubular adenoma found in a pregnant woman in 2015 [15]. A rare case of a pre-existing tubular adenoma with rapid tumour enlargement during pregnancy has also been reported [4]. Our patient was not pregnant and late presentation is thought to account for the uncharacteristically large size. Tubular adenomas are typically present for 2–12 months before a diagnosis is made [16]. In our case, the interval before presentation of 10 years is a remarkable outlier in the literature. The term “giant tubular adenoma” has been used before, but it is unclear what size constitutes a *giant* tumour. However, when the term has been used for fibroadenomas it describes those weighing more than 500 g, measuring more than 5 cm in size or that replace more than 80% of the breast volume [17]. We propose therefore using a similar threshold to categorise giant tubular adenomas of the breast. The presence of ulceration and fungation in association with lymph nodes was initially considered to be indicative of malignancy. However, multiple pre-operative core-needle biopsies and a meticulous search in the entire resection sample revealed no evidence of malignancy. The ulceration was thought to have resulted from pressure necrosis from this large expansile mass exacerbated by the burn. This phenomenon has been described before with giant fibroadenomas [18,19] and phyllodes tumour [20] and has been noted to cause a similar diagnostic dilemma. The solitary lymph nodes were assumed to have been reactive as a result of infection at the ulcer site. This was corroborated on histological examination. The choice of mastectomy for the treatment of the breast was influenced by the unprecedented size of the lesion, the paucity of normal breast tissue, the large ulcer and the lingering suspicion of malignancy. Dolmans et al performed mastectomy in a patient with a giant fibroadenoma because of concerns about the high ratio of affected breast to healthy breast [21]. However, Hille-Betz et al found that after simple tumour excision the remaining breast tissue expanded post-operatively, achieving excellent symmetry [22]. In a recent systematic review, Sosin et al recommended mastectomy only for unusual or recurrent cases [23]. Such radical surgery could conceivably have been avoided.

## 4 Conclusions

Maffucci’s syndrome is a rare syndrome which is characterised by the combination of enchondromatosis and vascular lesions. Tubular adenoma of the breast has not been previously reported in association with Maffucci’s syndrome. Future genome-wide association studies may uncover a genetic link between these conditions. Physicians should remember that large, rapidly growing benign breast tumours may cause ulceration and subsequent reactive lymphadenopathy that can lead to confusion with malignancy. Breast-conserving excision should be attempted even when the ratio of tumour to normal breast tissue is high.

## Ethical approval

Ethical approval for this publication has been exempted by our institution.

## Consent

Written informed consent was obtained from the patient for publication of this case report and accompanying images. A copy of the written consent is available for review by the Editor-in-Chief of this journal on request.

## Author’s contribution

Dennis Mazingi – case report design, subject research, data acquisition, consent and writing.

Chenesa Mbanje – case report design, writing, research and editing.

George C Jakanani – case report design, writing, research and editing.

Godfrey I Muguti – case report research and editing.

Valentine Mandizvidza – case report research and editing.

Shingi Bopoto – case report design, data acquisition, writing and editing.

## Registration of research studies

N/A.

## Guarantor

Dr Dennis Mazingi.

## Provenance and peer review

Not commissioned, externally peer-reviewed.

Nil.

## Declaration of Competing Interest

Nil.
